# Inhibition of p38 mitogen-activated protein kinase exerts a hypoglycemic effect by improving β cell function via inhibition of β cell apoptosis in db/db mice

**DOI:** 10.1080/14756366.2018.1477138

**Published:** 2018-10-04

**Authors:** Xiaowei Wei, Nan Gu, Nan Feng, Xiaohui Guo, Xiaowei Ma

**Affiliations:** Endocrinology Department, First Hospital, Peking University, Beijing, China

**Keywords:** p38 MAPK, SB203580, β cell function, endoplasmic reticulum stress, type 2 diabetes

## Abstract

The p38 mitogen-activated protein kinase (MAPK) pathway is involved in endoplasmic reticulum stress (ERS) and inflammation, which may play an important role in the pathogenesis of type 2 diabetes (T2DM). This study aimed to investigate whether p38 MAPK contributes to the pathogenesis of T2DM. 6-week-old female db/db mice were randomly assigned to Dmo and Dmi groups, and C57 mice were assigned as controls. The Dmi group was gavaged with the p38 MAPK inhibitor SB203580 for 9 weeks, and the effects on β cell dysfunction and apoptosis were investigated. db/db mice showed higher food intake, body mass, fasting glucose, and plasma insulin levels than C57 mice. After SB203580 administration, blood glucose was significantly lower. HOMA β and HOMA IR were improved. Islet mRNA expression levels of the ERS markers were lower. P38 MAPK inhibition reduced blood glucose and improved β cell function, at least in part by reducing β cell apoptosis.

## Introduction

β cell dysfunction and insulin resistance are the main pathogenic factors involved in the development of type 2 diabetes (T2DM)^1^. A progressive deterioration in β cell function is an essential component of T2DM^2^. Previous studies confirm that glucotoxicity, lipotoxicity, and oxidative stress all participate in β cell dysfunction and incite apoptosis^3^. All these factors lead to endoplasmic reticulum stress (ERS) and inflammation, which can co-exist and promote one another via shared pathways.

Mitogen-activated protein kinases (MAPKs) play pivotal roles in cell survival, apoptosis, proliferation, and differentiation. p38 MAPK (p38) is a MAPK family member that is involved in inflammation and stress responses. Pro-inflammatory cytokines, such as interleukin (IL)-1, IL-6, and tumor necrosis factor α (TNF-α), can upregulate p38 phosphorylation, thereby activating the kinase, and activated p38 further promotes inflammation by increasing transcription of pro-inflammatory factors. SB203580 is a p38 inhibitor that is widely used as a research tool. Inhibitors of p38 such as SB203580 block classical p38 pathways by inhibiting the α and β isoforms, thus down-regulating inflammation. Newer inhibitors have also been used to treat chronic obstructive pulmonary disease^4^, coronary heart disease^5^, hypercholesterolemia^6^, and other chronic inflammatory diseases in clinical research studies^7^.

Recent studies show that activation of the p38 pathway promotes β cell apoptosis through inflammatory responses and the ERS. Specifically, Song et al.^8^ showed that calcium-independent phospholipase A_2_β (iPLA_2_β), downstream of p38, is involved in islet β cell apoptosis in cultured INS-1 and isolated mouse islets. The p38 inhibitor PD169316 prevented thapsigargin-induced apoptosis of β cells by blocking p38 activation, while inhibition of p38 using SB203580 attenuated β cell apoptosis induced by the pro-inflammatory factors IL-1 β and interferon (IFN)-γ^9^^,^^10^. Finally, a study of the pathogenesis of diabetes in Akita mice found that the p38 pathway was involved in ERS-induced β cell apoptosis^11^.

Our preliminary studies showed that the rs2076878 of p38 is associated with insulin secretion (*p* < 0.05) and the risk of prediabetes (*p* = 0.016) in the Chinese Han population. In subsequent functional studies, rs2076878 was also shown to influence p38 mRNA expression (*p* = 0.034)^12^. Therefore, we hypothesize that p38 is involved in the pathogenesis of T2DM and administration of its inhibitor SB203580 could preserve islet β cell mass and function by attenuating ERS and inflammation.

In this study, the role of p38 and the effect of its inhibitor SB203580 on β cell function and apoptosis were investigated in db/db mice, an animal model of T2DM.

## Materials and methods

### Animals and procedures

Six-week-old female db/db mice (BKS.Cg-m+/+Leprdb) and C57BLKS/J mice were purchased from the Model Animal Research Center of Nanjing University. Mice were housed under controlled light (12 h light/12 h dark) and temperature conditions, and had free access to chow and water. All procedures were conducted in accordance with the guidelines on Animal Care and were approved by the Institutional Animal Care and Use Committee (IACUC) of Peking University Health Science Center (PUHSC).

After 1 week of acclimatization, six db/db mice and six C57 mice were euthanized and their pancreases were removed for islet isolation or immunohistochemical analysis. The remaining mice were divided into three groups: a C57 mouse control group (Con) (*n* = 18), a db/db SB203580 (LC Laboratories) gavage group (Dmi) (*n* = 18), and a db/db distilled water gavage group (Dmo) (*n* = 18). Mice in the Dmi group were administered with 15 mg/kg SB203580 daily by gavage, while Con and Dmo mice were administered with the same volume of distilled water. Food intake, body mass, fasting blood glucose (FBG), and fasting insulin (FI) levels were measured once a week. After a statistically significant difference was detected in FBG between the Dmi and Dmo groups, intraperitoneal glucose tolerance tests (IPGTTs) were performed on Dmi and Dmo mice at 2, 4, and 6 weeks. On day 3 after each IPGTT, six mice were picked randomly from each of the groups and euthanized for subsequent molecular biological and morphological assays.

### Measurements of physiological and biochemical indices

Mice were fasted overnight for 16 h and allowed to adapt to their environment for 5–10 min before further assays were performed. Body mass was obtained by weighing the mice in the fasting state using a balance. Blood glucose levels were measured using a blood glucose meter (Roche Corp., Germany) and ∼0.6 µl tail blood. Plasma insulin was assayed in plasma obtained from 30 µl blood samples collected in 0.5 ml EDTA tubes, using an Ultra-Sensitive Mouse Insulin ELISA Kit (Biofine Inc., Beijing, China).

### Intraperitoneal glucose tolerance test

For the glucose tolerance test, mice were fasted for 16 h and intraperitoneally injected with 10% glucose at 1 mg/g body mass. At 0, 30, 60, 90, and 120 min after injection, tail blood glucose levels were measured and 30 µl tail blood was collected for insulin assay.

### Immunofluorescence analysis

Pancreases were collected in sterile tubes, immediately frozen in liquid nitrogen, and then transported and stored at −80 °C. Five micrometer serial sections of frozen pancreas were obtained, and slides were fixed in acetone at 4 °C for 10 min and air-dried for 20 min. After three washes in phosphate-buffered saline (PBS), they were incubated in 0.1% triton X-100 for 10 min. After three further washes in PBS, the sections were blocked with 3% bovine serum albumin (BSA) for 1 h. They were then incubated in primary antibody (1:200 mouse anti-mouse insulin IgG solution, containing 1% BSA (Peking University Health Science Center, Biology Department, Beijing, China) overnight at 4 °C, exposed to room temperature for 30 min, washed, and then incubated at 37 °C in the dark with secondary antibody (donkey anti-mouse Cy5 IgG (Jackson Immunoresearch Laboratories Inc., West Grove, Pennsylvania, United States of America) for 60 min. Thereafter, slides were sealed with mounting medium containing DAPI (ZSGB-BIO, Inc., Beijing, China), and air-dried. Images were captured using an Olympus fluorescence microscope. Details of the detection method have been described elsewhere^13^.

### Islet isolation

Islets were isolated from pancreases by liberase (Roche Diagnostics) digestion, followed by separation on a discontinuous Ficoll gradient and manual stereomicroscopic selection, to exclude contaminating tissues.

### Real-time reverse transcriptase (RT)-PCR analysis

Total RNA was extracted from isolated islets using TRIzol Reagent (Invitrogen, USA). cDNAs were synthesized using a High-Capacity cDNA Reverse Transcription Kit (Applied Biosystems, USA) according to the manufacturer’s protocol. The primers used for RT-PCR are listed in Supplementary Material 1. Real-time reverse transcriptase (RT)-PCRs were performed using an ABI 7300 Real-Time PCR System (Applied Biosystems, USA) and SYBR Green master mixes (Applied Biosystems) according to the recommended protocol. mRNA expression was calculated using the comparative CT method (X_Test_/X_β-actin_** **= 2^−ΔΔCt^), using β-actin as the endogenous reference gene.

### Western blot analysis

Islets from mice of the same group that were sacrificed at the same time-point were pooled to obtain a single sample in sufficient quantity for western blotting. Islets were lysed in Radio-Immunoprecipitation Assay (RIPA) lysis buffer. Protein concentrations were measured by bicinchoninic acid (BCA) Protein Assay Kit (Biotime, Shanghai, China) according to the manufacturer’s recommended protocol. Aliquots containing 60 µg protein were separated by sodium dodecyl sulfate polyacrylamide gel electrophoresis (SDS-PAGE) using a 5% stacking gel and a 12% separating gel. Subsequently, proteins were blotted onto nitrocellulose membranes and probed with rabbit anti-p38 monoclonal antibody (Cell Signaling Technology, Inc.) or rabbit anti-phosphorylated p38 monoclonal antibody (Cell Signaling Technology, Inc.), followed by incubation with anti-rabbit IgG conjugated with horseradish peroxidase (ZSGB-BIO, Inc., Beijing, China). Proteins were detected by enhanced chemiluminescence.

### Statistical analysis

Data are expressed as mean ± SD. Significant differences among groups were identified by one-way analysis of variance and Turkey’s multiple comparisons test or by unpaired two-tailed Student’s *t*-test, using SPSS version 13.0. The area under the curve (AUC) for blood glucose or plasma insulin during an IPGTT was calculated using a trapezoidal method (AUC = 15 × 0 min +30 × 30 min +45 × 60 min +30 × 120 min). β cell function and insulin resistance index were evaluated using the Homeostasis Model Assessment (HOMA). HOMA β = 20 × [FI (mIU/L)/(FBG (mmol/L) − 3.5) (%)]. HOMA IR = FBG (mmol/L) × FI (mIU/L)/22.5. The islet area for each mouse was determined as the mean of the insulin positive areas of 10 consecutive frozen sections of the pancreas.

## Results

### Oral administration of SB203580 reduces blood glucose without affecting food intake, body mass, or plasma insulin

**Figure 1.**
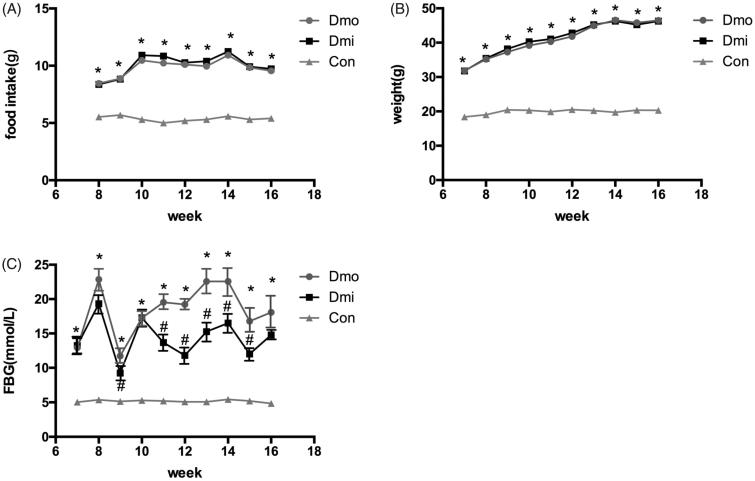
Metabolic features of the mice. (A) Food intake. (B) Body mass. (C) Fasting glucose levels (FBG). Dmo, vehicle-treated db/db mice; Dmi, SB203580-treated db/db mice; Con, C57 mice (*p < 0.05, Dmo or Dmi vs. Con; #*p* < 0.05, Dmi vs. Dmo).

**Figure 2.**
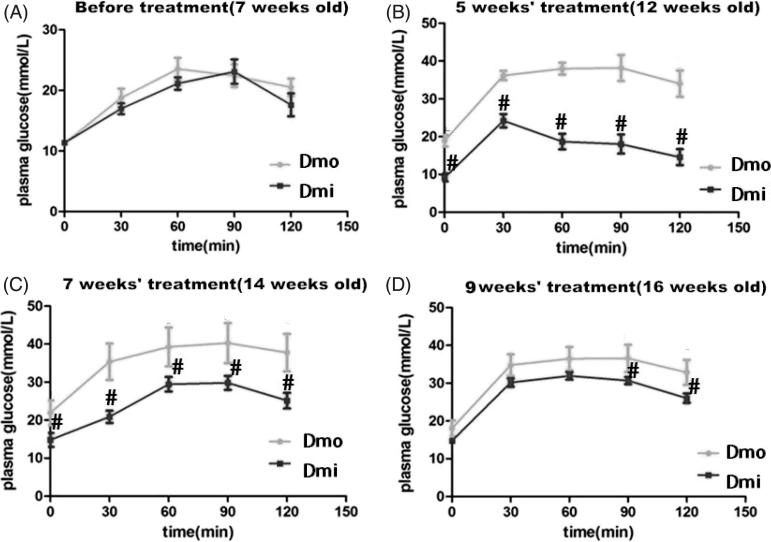
Blood glucose levels during intraperitoneal glucose tolerance tests (IPGTTs) in db/db mice throughout the experimental period. (A) Before administration of SB203580. (B) After 5 weeks’ administration of SB203580. (C) After 7 weeks’ administration of SB203580. (D) After 9 weeks’ administration of SB203580. Dmo, vehicle-treated db/db mice; Dmi, SB203580-treated db/db mice (#*p* < 0.05, Dmi vs. Dmo).

Female C57 mice and db/db mice administered with SB203580 or vehicle were monitored weekly for FBG, food intake, and body mass from the age of 7 weeks to identify any differences between db/db and C57 mice and the effects of SB203580. During the study, db/db mice showed higher food intake (Figure 1(A)), body mass (Figure 1(B)), and higher fasting glucose than C57 mice. SB203580 significantly reduced FBG from 5 weeks of treatment, but neither of the other parameters in db/db mice throughout the study (Supplementary Material 2). However, this reduction was not sufficient to normalize FBG to its level in C57 mice (Figure 1(C)). To further investigate the hypoglycemic effect of SB203580, IPGTT was undertaken. Consistent with the above finding, glucose tolerance was improved significantly in SB203580-treated db/db mice compared with vehicle-treated 12-, 14-, and 16-week-old mice, especially after 5 weeks of treatment (Figure 2).

### SB203580 administration improves β cell function and decreases insulin resistance in db/db mice

**Figure 3.**
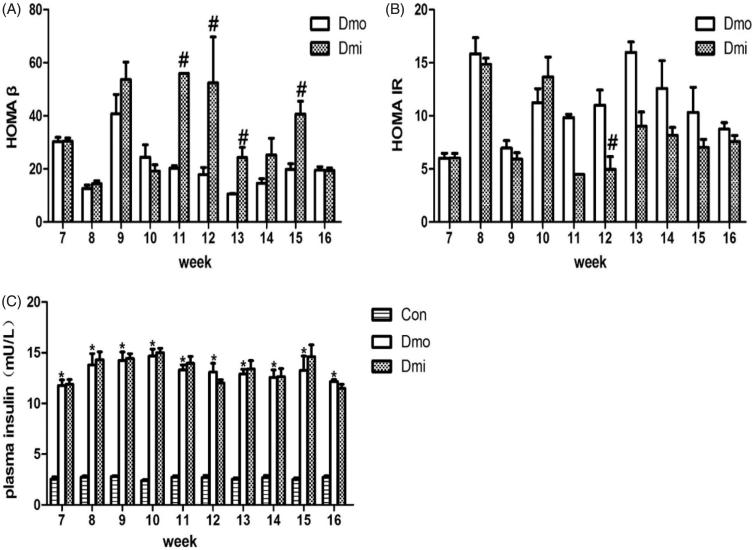
Fasting plasma insulin levels, HOMA β and HOMA IR of db/db mice. (A) HOMA β of db/db mice. (B) HOMA IR of db/db mice. (C) fasting insulin levels of mice. Con, C57 mice; Dmo, vehicle-treated db/db mice; Dmi, SB203580-treated db/db mice (#*p* < 0.05, Dmi vs. Dmo; *p < 0.05, Dmo or Dmi vs. Con).

Fasting insulin (FI) was measured along with FBG levels. As expected, FI was higher in T2DM mice to compensate for insulin resistance, but there were no significant differences between the Dmi and Dmo groups (Figure 3(C)). HOMA β and HOMA IR were then calculated to evaluate β cell function and insulin resistance. From 4 weeks (age = 11 weeks) after the start of SB203580 administration, HOMA β was significantly higher in SB203580-treated than vehicle-treated db/db mice, indicating that SB203580 treatment improved β cell function (Figure 3(A)). In addition, HOMA IR was significantly lower in SB203580-treated db/db mice than in vehicle-treated mice, but only on the fifth week of the intervention (Figure 3(B)).

### Expression of p38 MAPK and phosphorylated p38 MAPK in islets of C57 and db/db mice

**Figure 4.**
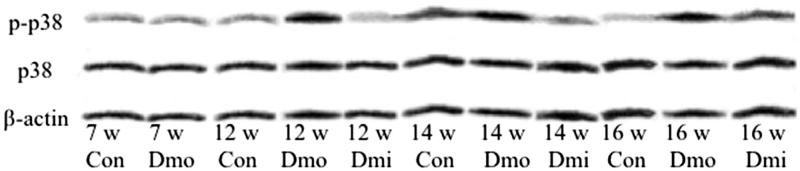
Protein expression of p38 and p-p38 in islets. p-p38, phosphorylated p38; Con, C57 mice; Dmo, vehicle-treated db/db mice; Dmi, SB203580-treated db/db mice.

SB203580 reduces p38 activity by inhibiting its phosphorylation. To determine the mechanism underpinning the improvement in β cell function observed in the treated mice, we examined the level of p38 phosphorylation by western blotting. No significant differences in protein expression of either total or phosphorylated p38 (p-p38) were found between 7-week-old C57 and db/db mice. Although the expression levels of p38 protein were not significantly different among the three groups, p-p38 was higher in untreated db/db mice (Figure 4**)** than C57 mice of the same age, and this difference was attenuated in SB203580-treated mice compared with vehicle-treated mice.

### Expression of BIP and CHOP in the islets of C57 and db/db mice

**Figure 5.**
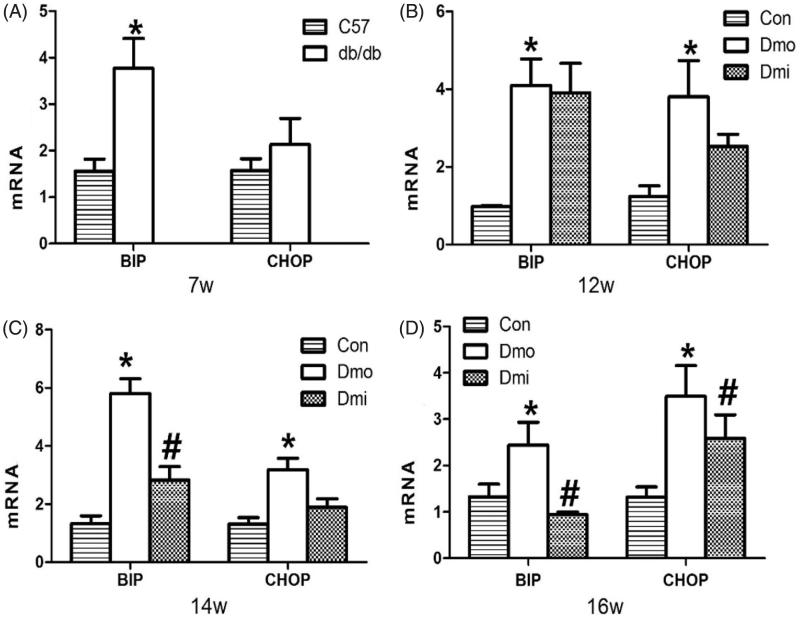
mRNA expression levels of BIP and CHOP in islets. Con, C57 mice; Dmo, vehicle-treated db/db mice; Dmi, SB203580-treated db/db mice (*, *p* < 0.05, Dmo vs. Con; #*p* < 0.05, Dmi vs. Dmo).

To determine whether p38 inhibition can modulate glucose homeostasis by inhibiting ERS in islets, we measured mRNA expression of BIP and CHOP in the mouse islets. Throughout the whole experimental period, BIP and CHOP expression levels were higher in vehicle-treated db/db mice than in C57 mice. Interestingly, mRNA expression in the SB203580-treated group was significantly lower than in the vehicle-treated db/db mice after 7 and 9 weeks of SB203580 administration (Figure 5). Thus, SB203580 administration ameliorated the diabetes-associated increase in expression of ERS mediators.

### Expression of Bcl-2 and Bax in the islets of C57 and db/db mice

**Figure 6.**
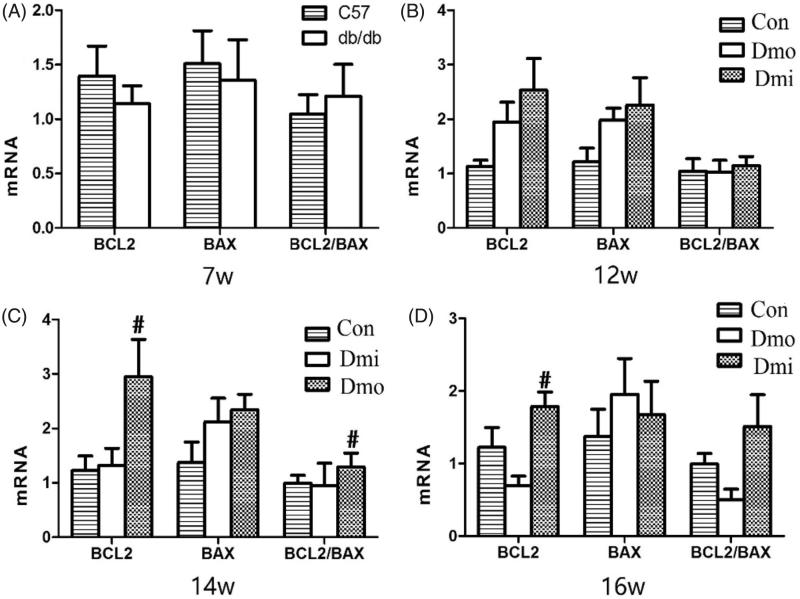
mRNA expression of Bcl-2 and Bax in islets. Con, C57 mice; Dmo, vehicle-treated db/db mice; Dmi, SB203580-treated db/db mice (*, *p* < 0.05, Dmo vs. Con; #*p* < 0.05, Dmi vs. Dmo).

The mRNA expression of Bcl-2, an important antiapoptotic protein, and Bax, an important proapoptotic protein, was measured by RT-PCR to evaluate the effect of SB203580 on apoptosis in the mouse islets. Bcl-2 mRNA levels were significantly higher in SB203580-treated 14- and 16-week-old db/db mice than in vehicle-treated mice, but there were no differences in Bax mRNA levels. The ratio of expression of Bcl-2 and Bax, which is negatively correlated with apoptosis, was then calculated, to estimate cellular apoptosis levels. The Bcl-2/Bax ratio was higher in SB203580-treated db/db mice than in vehicle-treated db/db mice, becoming significantly different after 7 weeks of inhibitor administration (Figure 6).

### β cell mass in C57 and db/db mice

**Figure 7.**
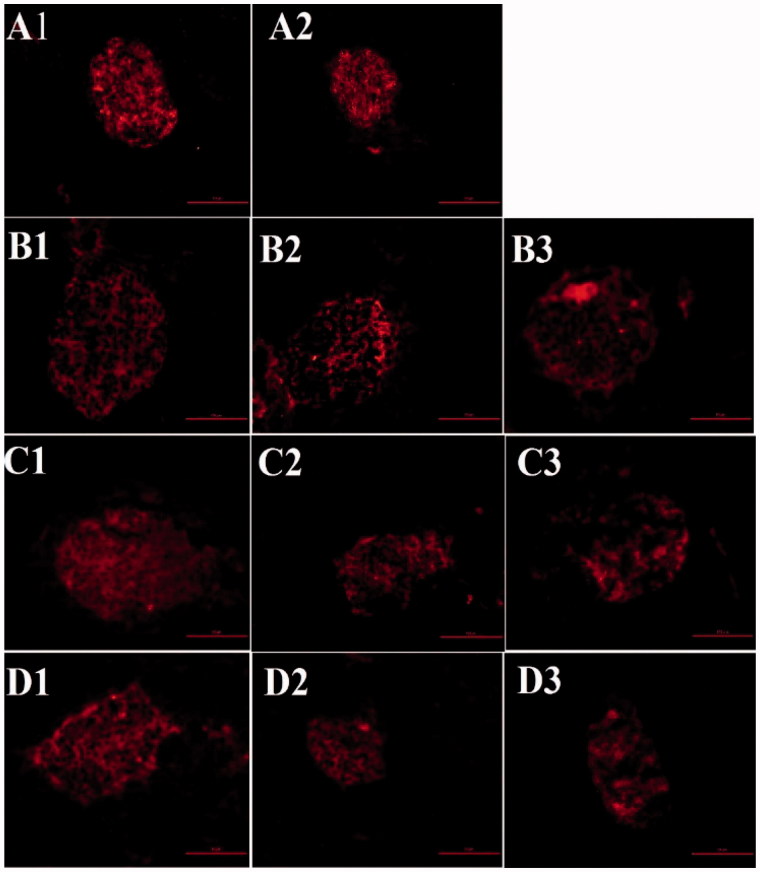
β cell area (insulin immunostaining, shown as a red color) of the pancreas in mice of various ages in each treatment group. A1, 7-week-old C57 mice; A2, 7-week-old db/db mice; B1, 12-week-old C57 mice; B2, 12-week-old vehicle-treated db/db mice; B3, 12-week-old SB203580-treated db/db mice; C1, 14-week-old C57 mice; C2, 14-week-old vehicle-treated db/db mice; C3, 14-week-old SB203580-treated db/db mice; D1, 16-week-old C57 mice; D2, 16-week-old vehicle-treated db/db mice; D3, 16-week-old SB203580-treated db/db mice.

β cell mass was estimated in islets separated from the pancreases of the mice by immunofluorescent staining for insulin. The untreated db/db mice showed a reduction in β cell mass during their lifetime (Figure 7). The β cell mass in untreated db/db mice was significantly lower than in C57 mice at 12 and 16 weeks of age. The SB203580-treated group tended to exhibit greater β cell mass, although this difference did not reach statistical significance (data not shown)

## Discussion

β cell dysfunction is a key pathogenic factor underpinning T2DM^1^. Inflammation and ERS use shared pathways that demonstrate mutual activation and lead to β cell dysfunction^14^^,^^15^. p38 MAPK plays a pivotal role in inflammation and has been associated with ERS responses^8^. Previous studies indicate that the p38 pathway promotes β cell apoptosis in T2DM by activating an inflammatory response and ERS^16^^,^^17^, and the inhibition of this pathway can protect β cells at the cellular level by alleviating inflammation and ERS^9^^,^^11^. Our preliminary genetic epidemiology studies showed that genetic variations in the p38 gene are associated with insulin secretion and the risk of prediabetes^12^^,^^18^. It is therefore important to identify the role of p38 and to determine whether it might be a potential target for the therapy of T2DM.

To validate our hypothesis, the role of p38 in the pathogenesis of T2DM was investigated on two levels. C57 mice were used as wild-type controls for the db/db mouse, a spontaneous model of T2DM. Metabolic parameters, β cell function and mass, and gene/protein expression (of p38, p-p38, ERS, and apoptosis) were measured in db/db mice during and after administration of a p38 inhibitor or vehicle.

In this study, untreated db/db mice possessed characteristics typical of T2DM, including obesity, hyperglycemia, and hyperinsulinemia. As expected, an increase in expression of active phosphorylated p38 was identified in the disease model, and mRNA expression levels of BIP and CHOP, key mediators of ERS pathways, were also higher. As a result, apoptosis (indicated by Bcl-2 and Bax expression) of β cells was affected. Islet area, measured following insulin immunostaining, was significantly lower in older mice, especially at 16 weeks of age. This series of changes strongly suggested that activation of the p38 pathway plays a role in the development of T2DM, at least in part by activating ERS pathways, thereby promoting apoptosis. To date, no relevant role for p38 in T2DM pathogenesis has been reported, either in animal models or in humans. The use of a p38 inhibitor has thus assisted us to better understand the role of p38 and the possible mechanisms of its effects.

In this study, the p38 inhibitor SB203580 significantly improved FBG and glucose tolerance independently of any reduction in body mass, which was consistent with the study by Yamaguchi K et al.^11^. Even though no significant differences in absolute levels of FI were detected between SB203580- and vehicle-treated db/db mice, SB203580-treated mice showed a significant increase in HOMA β compared with control db/db mice. Furthermore, the reduction in HOMA IR did not seem to be a major factor in the hypoglycemic effect of SB203580 because this was only detected during 1 week of the treatment. Therefore, the primary conclusion that can be drawn is that p38 inhibitor administration lowered blood glucose levels mainly by improving islet β cell function, independent of insulin resistance.

**Figure 8.**
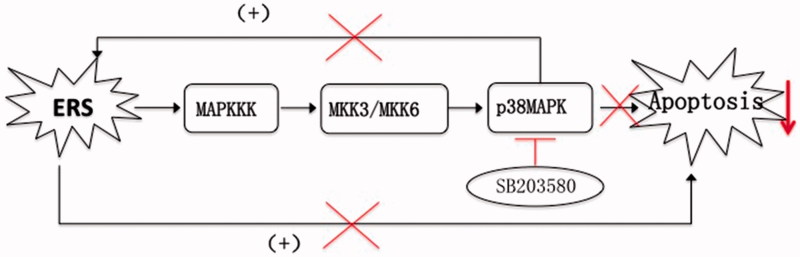
A schematic view of the mechanisms whereby the p38 inhibitor SB203580 may reduce β cell apoptosis. ERS leads to cellular apoptosis through activation of the p38 MAPK pathway. Activation of p38 can promote the activation of ERS pathways, thus inducing apoptosis. By contrast, the p38 inhibitor SB203580 attenuates activation of the p38 MAPK pathway, reducing apoptosis mediated through the p38 pathway, but also by inhibition of ERS, as part of a benign cycle.

To understand the effects of p38 inhibition on β cell function, the expression of key mediators in ERS pathways and markers of apoptosis was measured, along with islet cross-sectional areas. SB203580 efficiently inhibited p38 phosphorylation in this study, confirming the expected effect. Compared with untreated db/db mice, as FBG improved, SB203580-treated db/db mice showed lower mRNA expression of ERS markers (BIP and CHOP). Bcl-2/Bax and Bcl-2 mRNA expression, which are negatively correlated with apoptosis, increased simultaneously. β cell mass tended to increase, although this did not reach statistical significance, which may be due to the small sample size. All the above findings suggested that activation of p38 could promote the activation of ERS pathways, inducing cellular apoptosis. By contrast, p38 inhibition can reduce β cell apoptosis and protect β cell function, not only directly through the p38 pathway, but also at least partially by inhibition of ERS. These findings are summarized in Figure 8.

Insulin resistance and β cell dysfunction are the two main pathogenic mechanisms involved in the development of T2DM. This study of mice with spontaneously occurring T2DM showed that p38 MAPK indeed plays a role in the development of T2DM by affecting β cell function and apoptosis. The study also showed that p38 MAPK may be involved in the mechanism of insulin resistance, indicated by a change in HOMA IR, but because this was reduced only at one experimental time-point, further investigations are required.

Some limitations of the study should be acknowledged. Firstly, as the number of mice in each group was relatively small, the results need to be verified using larger numbers of animals. Secondly, the mRNA expression levels of key mediators were measured; protein levels should also be evaluated to enhance the physiological relevance of the study. In addition, some studies demonstrated that inhibition of the p38 pathway can lower blood glucose by improving insulin sensitivity in peripheral organs^19^^,^^20^, which the finding of a temporary SB203580-mediated improvement in HOMA IR is to some extent consistent with. Therefore, insulin tolerance testing and measurement of expression levels of mediators of insulin sensitivity in insulin target tissues should be carried out in a future study, to explore the role of p38 in insulin sensitivity.

In summary, this study demonstrated that p-p38 MAPK levels increased in the islets of T2DM db/db mice. SB203580, a p38 MAPK inhibitor, can lower blood glucose by improving β cell function, mediated through a reduction in β cell apoptosis. Activation or inhibition of the p38 MAPK pathway may be involved in the development or remission of T2DM by inducing or reducing β cell apoptosis and improving β cell function through both direct and indirect mechanisms. It is very important to explore the mechanisms of β cell damage in T2DM and to seek novel targets for the therapy of T2DM in this way.

## Supplementary Material

Supplementary_Material-r2.docx
