# Supplementary material for: Inhibition of p38 mitogen-activated protein kinase exerts a hypoglycemic effect by improving β cell function via inhibition of β cell apoptosis in db/db mice
Source: J Enzyme Inhib Med Chem. 2018 Oct 4;33(1):1494–500. doi: 10.1080/14756366.2018.1477138 (PMC6179047; doi:10.1080/14756366.2018.1477138)
Supplement: Supplementary_Material-r2.docx [file IENZ_A_1477138_SM1433.docx]

**Supplementary Material**

**Inhibition of p38 mitogen-activated protein kinase exerts a hypoglycemic effect by improving β cell function via inhibition of β cell apoptosis in db/db mice**

**Short title: P38 inhibitor inhibits β cell apoptosis**

Xiaowei Wei^1^, Nan Gu^1^, Nan Feng^1^, Xiaohui Guo^1^, Xiaowei Ma^1^*

**Affiliations:** ^1^Endocrinology department, First Hospital, Peking University, Beijing ,China (Xiaowei Wei, Nan Gu, Nan Feng, Xiaohui Guo, Xiaowei Ma*)

**Corresponding author**: Xiaowei Ma email: [**mxiaowei1967@sina.com**](mailto:xiaowei.ma@pkufh.cn); Fax: 86-10-66552395, Tel.: 86-10-83575103

**Supplementary Material 1**

**Sequences of primers used for RT-PCR**

| **Gene** | **Primer sequences** |
| --- | --- |
| ***β-actin*** | \| Forward: CCCTAAGGCCAACCGTGAA  Reverse: CACAGCCTGGATGGCTACGT \| \| --- \| |
| ***Bip*** | Forward: GAGACTGCTGAGGCGTATTTGG  Reverse: CGCTGGGCATCATTGAAGTA |
| ***Chop*** | \| Forward: TCGGACGCACTTGGAATGA  Reverse: AACCACCTTGAATGGCAAGAA \| \| --- \| |
| ***Bcl-2*** | Forward: GGCATCTGCACACCTGGAT  Reverse: TCAAACAGAGGTCGCATGCT |
| ***Bax*** | Forward: TGGAGCTGCAGAGGATGATTG  Reverse: TGCCATCAGCAAACATGTCA |

**Supplementary Material 2**

**Metabolic features of the mice**

| Weeks |  | Con |  |  | Dmo |  |  | Dmi |  |
| --- | --- | --- | --- | --- | --- | --- | --- | --- | --- |
|  | Food intake (g/day) | Weight (g) | FBG (mmol/L) | Food intake (g/day) | Weight (g) | FBG (mmol/L) | Food intake (g/day) | Weight (g) | FBG (mmol/L) |
| 7 |  | 18.40±1.14 | 5.03±0.39 |  | 31.85±1.76* | 12.93±3.86* |  | 31.76±1.65 | 13.23±3.58 |
| 8 | 5.53±0.34 | 19.03±1.16 | 5.37±0.53 | 8.46±0.21* | 35.19±1.74* | 22.86±6.05* | 8.37±0.41 | 35.43±1.66 | 19.33±5.04 |
| 9 | 5.7±0.25 | 20.42±1.14 | 5.13±0.42 | 8.88±0.19* | 37.29±1.65* | 11.72±3.17* | 8.83±0.49 | 38.18±1.26 | 9.23±3.18# |
| 10 | 5.3±0.41 | 20.32±1.04 | 5.28±0.43 | 10.47±0.33* | 39.17±1.81* | 17.26±4.70* | 10.92±0.12 | 40.25±2.29 | 17.29±4.63 |
| 11 | 5.0±0.18 | 19.90±0.45 | 5.20±0.36 | 10.23±0.98* | 40.31±1.98* | 19.54±3.85* | 10.84±0.11 | 41.08±2.39 | 13.68±3.80# |
| 12 | 5.2±0.22 | 20.50±0.99 | 5.07±0.40 | 10.10±0.49 | 41.81±2.47* | 19.22±2.39* | 10.27±0.51 | 42.83±2.48 | 11.81±3.67# |
| 13 | 5.3±0.17 | 20.23±0.85 | 5.08±0.53 | 9.96±0.18* | 44.92±2.04* | 22.58±4.19* | 10.4±0.51 | 45.22±2.94 | 15.27±3.58# |
| 14 | 5.6±0.87 | 19.72±0.99 | 5.43±0.50 | 10.92±0.21* | 46.56±2.16* | 22.57±4.82* | 11.24±0.35 | 46.26±3.25 | 16.52±3.93# |
| 15 | 5.3 | 20.35±1.20 | 5.20±0.14 | 9.84 | 45.82±1.73* | 16.78±3.17 | 9.92 | 45.23±1.68 | 12.00±1.73# |
| 16 | 5.4 | 20.30±0.99 | 4.85±0.07 | 9.56 | 46.48±1.88* | 18.08±4.65 | 9.73 | 46.28±1.90 | 14.78±1.37 |

Data are mean ± SD. Treatment groups were compared using independent t-tests. Dmo, vehicle-treated db/db mice; Dmi, SB203580-treated db/db mice; Con, C57 mice (*, p<0.05, Dmo or Dmi vs. Con; #, p<0.05, Dmi vs. Dmo)
